# Disparities in child health in the Arab region during the 1990s

**DOI:** 10.1186/1475-9276-7-24

**Published:** 2008-11-20

**Authors:** Marwan Khawaja, Jesse Dawns, Sonya Meyerson-Knox, Rouham Yamout

**Affiliations:** 1 Center for Research on Population and Health, American University of Beirut, Van Dyck Hall, Box 11-0236, Riad El-Solh1107 2020, Beirut, Lebanon

## Abstract

**Background:**

While Arab countries showed an impressive decline in child mortality rates during the past few decades, gaps in mortality by gender and socioeconomic status persisted. However, large socioeconomic disparities in child health were evident in almost every country in the region.

**Methods:**

Using available tabulations and reliable micro data from national household surveys, data for 18 Arab countries were available for analysis. In addition to infant and child mortality, child health was measured by nutritional status, vaccination, and Acute Respiratory Infection (ARI). Within-country disparities in child health by gender, residence (urban/rural) and maternal educational level were described. Child health was also analyzed by macro measures of development, including per capita GDP (PPP), female literacy rates, urban population and doctors per 100,000 people.

**Results:**

Gender disparities in child health using the above indicators were less evident, with most showing clear female advantage. With the exception of infant and child survival, gender disparities demonstrated a female advantage, as well as a large urban advantage and an overall advantage for mothers with secondary education. Surprisingly, the countries' rankings with respect to disparities were not associated with various macro measures of development.

**Conclusion:**

The tenacity of pervasive intra-country socioeconomic disparities in child health calls for attention by policy makers and health practitioners.

## Background

Scholarly and policy interest in health disparities and in the relationship between inequality and health status has increased dramatically over the past few decades [1-6], but most of it focused on the developed countries [7-11]. Similarly, while research linking poor outcomes in child health to poverty and inequities was growing, much was still based in the developed countries [12-14]. However, the burden of child mortality and morbidity falls most heavily on the developing world [15-17]. As Black et al pointed out, "More than 10 million children die each year, most from preventable causes and almost all in poor countries" [16].

In the Arab region, several studies documented gender disparities in health, including female disadvantage in survival chances and access to health care for children and older adults [18-23]. However, empirical evidence regarding the link between socioeconomic (or social class) disadvantage and child health in the region remains tenuous.

Arab countries have made considerable progress in the past few decades in improving the health and wellbeing of their people. Infant mortality rates (IMR) for the region witnessed an impressive decline of about 69% from 1970 to the present, a sharper decrease than in most other developing regions [21,24]. However, despite such progress, or perhaps because of it, disparities within and across countries remained and may have even increased over time [21,25].

Numerous socioeconomic factors play a role in determining child health, and the Arab region demonstrates considerable disparities in terms of socioeconomic and human development both within and across countries. Although intra-country socioeconomic disparities are more difficult to measure, in Tunisia – ranked by the UNDP as having a medium-level of human development – the richest 10% of the country's households absorbed 47.3% of all income/consumption, compared to only 2.3% by the poorest 10% [18].

Within the literature that exists, inter-country infant and child mortality disparities in the region have been specifically linked to wealth and socioeconomic status. Infant mortality for the region was found to be inversely correlated to female and male literacy rates, as was annual GNP per capita, the proportion of the population with safe drinking water, and adequate access to sanitation facilities [26].

Moreover, excess female child mortality has been prevalent in most if not all Arab countries, ranging from 10 to 30% [21]. For countries with low levels of mortality, male children will normally have higher mortality rates than females [27]. Thus, gender parity in child mortality indicates female disadvantage.

Health is more than survival and mortality, however, and should include consideration of factors that capture the deterioration of health, such as nutritional status and diseases, as well as measures that protect children's health, like immunization coverage. According to the WHO's Global Database on Child Growth and Malnutrition, child malnutrition in the region, as measured by stunting, has decreased on the whole since 1980 [28]. However, more than one in six children still suffered from malnutrition across the region, and one in 13 from severe malnutrition [21].

In the Arab region, intra-country socioeconomic disparities have also been shown to affect childhood nutritional status. Inequities like low household income, low maternal level of education, or residence in rural or squatter areas were shown in various studies to negatively impact child health indicators like under- or mal-nutrition, wasting or stunting in different Arab countries [21,29-32].

This article provides a descriptive portrait of disparities in child health in 18 countries in the Arab region during the 1990s, using available tabulations and reliable micro data from national household surveys. Although descriptive, the paper attempts to answer three questions: (1) what was the extent of within-country disparities in child health in the region in the 1990s? (2) of the gender and socioeconomic disadvantages facing Arab children, which is greater?, and (3) is there an association between levels of health disparities and socioeconomic development of countries? An assessment of existing disparities in child health and the factors associated with them is an important undertaking if we are to arrive at appropriate remedial interventions designed to improve equity in health.

## Methods

### Data sources

This study was based on both survey data on child health indicators and macro-level data on the socioeconomic development of the countries under examination. In both cases, we relied primarily on secondary sources or readily available, published reports. Unlike previous studies using largely UN estimates or data from small samples, our main sources of data on child health were population-based household surveys conducted in 18 Arab countries during the 1990s. The majority of the surveys were completed during 1995–1998. The exceptions were Mauritania (1990/92) [33], Algeria (1992) [34], Sudan (1992–93) [35] and Syria (1993) [36]. The surveys were from the following sources: DHS (Egypt [37], Jordan [38], Yemen [39]), PAPChild (Algeria [34], Lebanon [40], Libya [41], Mauritania [33], Morocco [42], Sudan [35], Syria [36], Tunisia [43]), Gulf Family Health Surveys (Bahrain [44], Kuwait [45], Oman [46], Qatar [47], Saudi Arabia [48], UAE [49]) and the PCBS Health Survey (Occupied Palestinian Territories) [50].

All of these national surveys relied on fairly standardized instruments and similar methodologies, thus enhancing comparability of data between countries and over time. Table 1 provides information about sample characteristics of all the surveys used here. In the surveys, women of reproductive age were asked the same questions about their reproductive history (dates of birth and age of death of all their live born children). Furthermore, women (or care givers) were asked questions about the health and nutritional status of their children aged less than 5 years.

**Table 1:** Summary of data sources, Arab countries

Country	Year of survey	Source	Number of households	Number of children under-five
Algeria	1992	PAP Child	6133	5092
Bahrain	1995	GFHS	4166	3120
Egypt	1995	DHS	16957	-
Jordan	1997	DHS	7335	-
Kuwait	1996	GFHS	3673	3514
Lebanon	1996	PAP Child	4600	2156
Libya	1995	PAP Child	6312	5348
Morocco	1990	PAP Child	5686	6000
Mauritania	1991–92	PAP Child	6717	4971
Occupied Palestinian Territory	1995–96	Health Survey	6204	6169
Oman	1995	GFHS	6103	9033
Qatar	1998	GFHS	4207	3775
Saudi Arabia	1996–97	GFHS	10510	10831
Sudan	1992–93	PAP Child	5320	4585
Syria	1993	PAP Child	5998	4352
Tunisia	1994–95	PAP Child	6085	3679
United Arab Emirates	1995	GFHS	5822	6285
Yemen	1997	DHS	10701	-

Data for national-level demographic and socioeconomic indicators were from the Human Development Report (1999) [51], and the UN PRED (2002) [24] and WHO (2002) [52] cross-national databases, except for Palestine. The 1999 HDR provided national level-level data from 1997 in the areas of real per capita GDP (PPP) and female literacy rates. UN PRED, a machine-readable database prepared by the Population Division of the United Nations Secretariat, provided data on national percentages of urban population for 1995. The number of doctors per 100,000 people in 1995 was obtained from the WHO database. Corresponding data for the Palestinian areas were obtained directly from the Palestinian Central Bureau of Statistics in Ramallah, West Bank for 1997–1998 [53].

All the indicators obtained were for the mid-1990s, or otherwise chosen based on their proximity to the year in which the survey for a given country was conducted. When we could not obtain macro data for the same year of the survey date, we used available data for the year closest to the survey date.

### Measures

Several child-health related measures were used. Mortality was measured by the IMR (the number of infant deaths per 1,000 live births) and the under-five mortality rate (U5MR, defined as the number of deaths of children under-five years per 1,000 live births).

Child nutritional status was measured by stunting and wasting. Stunting was defined as the proportion of children below -2 standard deviations from the median height-for-age of the reference of the NCHS/CDC/WHO international reference population, and signifies long-term or chronic deprivation. Stunting can be correlated with poor feeding practices, repeated infections and low socioeconomic status [27]. Wasting, an indicator of acute malnutrition [27], was defined as the proportion of children below -2 standard deviations from the median weight-for-age of the same international reference population.

Child morbidity was measured by Acute Respiratory Infection (ARI), as reported by mothers or primary care givers. ARI was measured by asking mothers about symptoms of ARI within 14 days preceding the survey. Preventive care for children was captured by immunization coverage. According to UNICEF, in the 1990s a child of 12–23 months was considered fully covered if s/he received the following vaccinations: BCG, measles, and three doses of DPT-Polio (Child Info 2007) [54].

In addition to gender, two socioeconomic indicators were also used to capture disparity: Place of residence (rural/urban) and mother's level of education (illiterate, primary level, or secondary and above). Several country-levels measures of socioeconomic developments were also used, including real GDP per capita in $US adjusted for purchasing power parities (PPP), female literacy rates, proportions of the population in urban areas, and number of medical doctors per 100,000 people.

### Analysis

Within-country disparities in child health by gender, residence (urban/rural) and maternal educational level were first described. Since the disparities discussed here were binary, simple disparity ratios (expressed per 100 persons) were calculated for each health outcome. For example, sex ratios of infant mortality were calculated for gender disparity in mortality for each country, indicating the number of female deaths per 100 males. Here, values exceeding 100 would indicate female disadvantage. Similar disparity ratios were computed for rural-urban residence and maternal education.

A summary measure of each disparity was then computed across countries in order to assess the associations between disparities and levels of socioeconomic development. First, each of the child health indicators described here was ranked in descending order to compare relative standings for each disparity in the region. Second, an average ranking score was obtained for wasting, stunting, immunization, and disease (ARI). Third, ranking scores for mortality were similarly created using solely disparity ratios for infant mortality. Finally, an overall ranking for each disparity was created by averaging the mortality ranking scores and the other child health scores.

Bivariate associations between the three disparities (in terms of rankings) and levels of socioeconomic development were then examined using Spearman's ranking correlation coefficients.

## Results

With the exception of child survival, gender disparities in child health demonstrated a female advantage. Although female deaths were well below those for males in nearly every country, the gap was not large enough when compared to a European standard. On the other hand, socioeconomic disparities in child survival and other child health indicators generally showed a large urban advantage, as well as an overall advantage for mothers with secondary education.

An urban advantage was evident in child survival, wasting, stunting and ARI, but not in immunization. Disparities by maternal level of education indicated an advantage for mothers with at least secondary education for child survival, fewer stunted and wasted children, and a greater immunization rate. There was no similar advantage to maternal education for ARI.

### Gender disparities

Estimates showed a consistent gender gap in IMR and U5MR (Table 2). Jordan had the lowest female disadvantage in infant mortality, with 68 female infants dying per 100 males. All countries under consideration showed a female advantage in both indicators, with the exception of Egypt (IMR 101 and U5MR 107) and Saudi Arabia (IMR 103).

**Table 2:** Disparity by Gender for Infant Mortality Rate, Wasting, Stunting, Acute Respiratory Infections, and Immunization Status, Arab countries

	IMR	U5MR	Wasting	Stunting	ARI	Immunization
	
Country	Female deaths for 100 male	Female deaths for 100 male	Females wasted for 100 male	Females stunted for 100 male	Females with ARI for 100 male	Males immunized for 100 female
Algeria	72	78	100	104	93	100
Bahrain	87	82	100	95	95	100
Egypt	101	107	96	92	93	101
Jordan	68	79	118	91	75	-
Kuwait	89	92	83	92	99	103
Lebanon	98	94	93	94	97	99
Libya	80	86	100	85	101	100
Mauritania	84	-	96	100	87	95
Morocco	87	94	85	93	98	100
Occupied Palestinian Territory	79	80	81	116	74	-
Oman	90	93	84	98	91	100
Qatar	80	94	-	-	94	-
Saudi Arabia	103	89	81	84	91	100
Sudan	90	85	110	99	98	100
Syria	84	90	91	93	81	101
Tunisia	81	89	123	101	104	100
United Arab Emirates	72	74	91	91	88	-
Yemen	81	89	88	98	92	101

However, the absolute equality in mortality and morbidity between the sexes is not necessarily and an indicator of gender parity, owing to the biological difference between the two sexes. It has been the practice to compare prevailing sex-differentials in mortality rates to a standard such as a model life table or a European one [22]. The average of mortality rates for both infants and children under five taken from 23 European life tables of the 1990s showed 80 female deaths for 100 male [22]. Taking this average as a standard, only three from the 18 Arab countries surveyed showed a female advantage in IMR; for U5MR, only the UAE had a real female advantage, at 74 female deaths. Twelve countries showed an excess female mortality in both IMR and U5MR.

From the 17 countries with data available for gender differences in child nutritional status, 13 countries had higher rates of stunting for male than female children. Palestine, however, showed a clear female disadvantage. There was also a clear male disadvantage with regard to wasting in the region. The average for wasting in the 17 countries was 95 females for 100 males, with 9 countries falling below that average. Only Jordan, Sudan and Tunisia had significantly higher rates of female child wasting.

Likewise, there was a male disadvantage in ARI in the region, with an average of 91 females with ARI for 100 males. Excepting Tunisia and Libya, all surveyed countries had higher rates of ARI symptoms for male children than for female children.

Available data for 13 countries did not show disparities between female and male children for immunization ratios, excepting Mauritania, which had a male disadvantage.

### Socioeconomic disparities

For child survival, data were available only by rural-urban residence, with no estimates for child survival by maternal level of education. Table 3 shows that child mortality rates were significantly higher in rural areas than urban areas. The largest urban-rural gaps in IMR were found in Tunisia and Morocco, with 226 and 194 rural deaths for 100 urban, respectively. For U5MR, there was again a regional urban advantage, with the largest advantage found in Tunisia, Morocco and Egypt.

**Table 3:** Disparity by rural-urban residence for infant mortality, child mortality, wasting, stunting, Acute Respiratory Infections, and immunization, Arab countries

	IMR	U5M	Wasting	Stunting	ARI	Immunization
	
Country	Number of rural deaths per 100 urban	Number of rural deaths per 100 urban	Number of rural children wasted for 100 urban	Number of rural children stunted for 100 urban	Number of rural children with ARI for 100 urban	Number of urban children immunized for 100 rural
Algeria	167	161	118	169	90	104
Bahrain	-	-	-	-	-	-
Egypt	170	180	96	151	98	102
Jordan	146	146	117	214	83	-
Kuwait	-	-	-	-	-	-
Lebanon	-	-	-	-	-	-
Libya	147	132	132	130	84	101
Mauritania	117	-	146	95	148	125
Morocco	194	204	125	172	63	103
Occupied Palestinian Territory	-	-	88	107	79	-
Oman	128	129	120	121	85	99
Qatar	-	-	-	-	-	-
Saudi Arabia	152	135	88	106	91	96
Sudan	113	121	129	169	93	126
Syria	117	112	86	106	59	97
Tunisia	226	231	111	225	74	102
United Arab Emirates	128	145	91	108	90	97
Yemen	124	134	132	138	103	104

In terms of stunting and wasting, there was a consistent urban advantage shown. Thirteen of the 14 countries with data on stunting and wasting showed an urban advantage for stunting, with the exception of Mauritania. Rural stunting was particularly high in Tunisia and Jordan. There was also a clustering of rural disadvantage in North Africa. In wasting, of the 14 countries that had data available on urban-rural differences, 9 showed higher rural rates of wasting, with the highest rural disadvantage found in Mauritania (146 rural per 100 urban). With the exception of Egypt, all North African countries included here showed rural disadvantage in wasting.

However, there was an urban disadvantage in ARI across the Arab region, with only Mauritania and Yemen showing an urban advantage. Syria had the most pronounced urban disadvantage for ARI, followed by Morocco, Tunisia and Palestine.

There were no major disparities in immunization coverage by the place of residence. The rural areas of Sudan and Mauritania, however, were more disadvantaged than the other Arab states.

Of the 16 countries with data on the rates of stunting and wasting by mother's level of education, the vast majority of countries showed a significant disadvantage of illiterate mothers when compared to mothers with at least a secondary education (Table 4). Estimates for 16 countries all showed an educational advantage for stunting, and 12 show one for wasting. For stunting, the largest educational advantages were found in Morocco (450) and Jordan (322). For wasting, the highest educational advantage was shown in Yemen (176), followed by Oman, Syria and Mauritania. Kuwait (61) and Saudi Arabia (89), however, showed a clear educational disadvantage for wasting.

**Table 4:** Disparity by maternal level of education for wasting, stunting, Acute Respiratory Infections, and immunization status, Arab countries

	Wasting	Stunting	ARI	Immunization
	
Country	Number of children of illiterate mothers wasted for 100 with secondary-level mothers	Number of children of illiterate mothers stunted for 100 with secondary-level mothers	Number of children of illiterate mothers with ARI for 100 with secondary-level mothers	Number of children of secondary mothers immunized for 100 illiterate mothers
Algeria	98	188	-	105
Bahrain	-	-	-	98
Egypt	116	148	110	105
Jordan	151	322	103	-
Kuwait	61	174	-	110
Lebanon	141	248	77	115
Libya	129	158	61	102
Mauritania	159	107	-	92
Morocco	123	450	56	102
Occupied Palestinian Territory	100	202	67	-
Oman	163	160	-	99
Qatar	-	-	-	118
Saudi Arabia	89	127	-	98
Sudan	141	296	77	145
Syria	160	173	85	110
Tunisia	121	1835	61	103
United Arab Emirates	112	143	-	113
Yemen	176	290	143	111

There was no apparent disparity by mother's education and frequencies of ARI symptoms. It should also be noted that data were available for only 10 countries; reliable data were lacking to assess disparities by mother's education for all of the wealthy Gulf States, as well as Algeria and Mauritania.

Significant disparities by the educational levels of mothers were observed for immunization coverage. Twelve countries showed a disadvantage of illiterate mothers compared to mothers with at least a secondary education. The highest disadvantage was found in Sudan (145). Of the four countries with a slight educational disadvantage, three were located in the Gulf (Bahrain: 98, Oman: 99, and Saudi Arabia: 98).

### Disparities and socioeconomic development

Table 5 presents the ranking of countries with respect to the three measures of disparity in child health. The three top ranked countries in child health disparities were UAE, Saudi Arabia and the OPT, and the lowest ranked countries were Bahrain, Qatar and Tunisia. Two other general trends were documented. First, there were apparent disagreements between the three different rankings, that is, countries with high scores on gender disparity may not necessarily rank high on residence or educational disparity. Second, there was a lack of correspondence between rankings of disparities on the one hand and income or regional groupings (e.g., Gulf, North Africa) on the other.

**Table 5:** Average rankings by form of disparity, Arab countries

Country	Gender	Rural-Urban residence	Maternal education
Algeria	7	7	8
Bahrain	13	-	-
Egypt	13	12	4
Jordan	2	7	16
Kuwait	6	-	2
Lebanon	16	-	9
Libya	7	7	6
Mauritania	10	3	12
Morocco	10	11	13
Occupied Palestinian Territory	3	-	4
Oman	7	4	14
Qatar	13	-	-
Saudi Arabia	7	4	2
Sudan	18	6	9
Syria	4	1	11
Tunisia	16	12	7
United Arab Emirates	1	2	1
emen	5	7	15

None of the disparities was significantly associated with any measure of development at the national level as judged by Spearman's rank correlation coefficients. Although all the correlation coefficients were generally weak, there were some noteworthy differences between disparities. For gender disparity, the associations with both female literacy (r_s _= -0.163) and the number of physicians (r_s _= -0.197) were rather weak, and there were no associations with urbanization or GDP per capita. The associations with educational disparity were also weak, but generally stronger, with coefficients ranging from -0.27 for number of physicians to -0.36 for GDP per capita. Patterns of associations with rural-urban residence were different, with a similar negative association with GDP per capita (r_s _= -0.19), urbanization (r_s _= -0.19) and female literacy (r_s _= -0.16). Unlike previously, there was no association between rural-urban disparity and the number of physicians (r_s _= -0.068).

## Discussion

According to the literature, income remains worldwide as the most important determinant of child survival and wellbeing across and within countries [55-57]. A recent study on the socioeconomic determinants of infant mortality in 152 countries reported that GNI/capita, young female illiteracy, and income equality (as measured by the Gini index) were significant determinants of IMR; for middle-income countries, income equality was an independent predictor [5]. Shawky [26] showed that in the Arab region a significant positive association existed between the level of income and an improvement in child health indicators.

Although the data did not show an absolute excess mortality of girls – rather seeming to indicate a male disadvantage – a comparison of gender disadvantage between the Arab countries and the European ones (used as a standard) showed that there was an excess female infant and under-five mortality in the region. Our findings correspond to those reached by Yount [22] in her study of sex differentials of child survival in the Middle East, with historical Northwest European life tables used as a standard. Our findings also correlated with those by Arnold [27]. In his review of Demographic and Health Surveys from 26 developing countries (1986–89), Arnold found that the male infant mortality rate in 25 countries averaged to 19% higher than that of female infants; the rates for the Arab states, however, were much lower. Previous authors have linked a disparity in child survival indicators to inequality in seeking health services, disparity in food allocation and care and unequal treatment of boys and girls by health care providers [19,23], all within a cultural context of son preference [20].

Female advantage in child nutritional status was documented. This can be explained by the biologically higher susceptibility of boys to diseases such as diarrhea and ARI, and their consequent failure to thrive. The male-female ratio in nutritional status rates should also be quantified in comparison to a standard, but such a standard was difficult to establish empirically. As cited by Arnold [27], in a 1990 UNICEF study of the wasting rates of boys and girls in 39 countries, the wasting of boys was found to be 1.3 times higher than that of girls. Our results showed that female advantage in nutrition in the Arab region was not as great. In particular, the Tunisian rate of wasting in girls (123/100) and Palestine's 116 stunted girls to 100 boys indicated a need to revise child nutritional policy.

Male disadvantage in ARI morbidity was also well documented. A male-female ratio of 1.25 has been reported in literature [58]. The average male-female ratio for ARI in Arab region was lower, at 1.1. This difference might have resulted from boys receiving favoured attention to their illnesses. This favoured care was quantified by Ahmed [19] in a study establishing that the ratio for ARI was significantly in favour of boys as compared to girls. Given the established male disadvantage, the rates of Libya and Tunisia favouring boys (101/100 and 104/100 respectively), are of concern.

The absence of gender disparity in immunization coverage was reported in the literature [59]. This achieved parity can be attributed to the generalization of immunization programs by most governments in Arab countries [59].

For disparity by place of residence, the levels in child mortality showed significantly higher rates in rural areas, as would be expected. The same trend was found in the nutritional status of children. Children living in rural environments showed a disadvantage in all indicators except for ARI. The urban penalty in ARI might have resulted from the higher rates of pollution in urban areas relative to rural places. This overall health disadvantage of rural populations in the Arab region was perhaps due to the lower socioeconomic status in rural settings that limit access to health care and adequate nutrition. However, it was difficult to separate these effects due to the lack of requisite micro data. There was no disparity by place of residence for immunization rates, again as a result of governmental policies in the region encouraging universal immunization coverage [60].

A significant disadvantage of illiterate mothers compared to mothers with at least secondary education was seen in wasting, stunting and vaccination rates. The disparities were higher than those found for gender and by place of residence. Although the benefits of literate mothers for children's health are well established, higher levels of education in mothers have also been linked to higher incomes [61]. However, an education disadvantage was indicated in some Gulf States for wasting (Kuwait and Saudi Arabia) and for immunization (Bahrain, Oman and Saudi Arabia). A possible explanation of this finding could be the involvement of foreign domestic workers in care provision to the children of highly educated mothers [62].

Overall, this paper provided evidence of pervasive inequalities in child health in the Arab region. Although the findings point to an apparent female advantage in mortality rates and nutritional status during the 1990s in the region, these rates were well below what would be expected using a European standard rates indicating persisting female disadvantage. This persistent female disadvantage is perhaps due to persisting discriminatory practices against female children in the region. Rural residence and illiteracy were clearly markers of disadvantage in almost any measure of child health used in this study. It also appears that the gender gap was less important than that based on socioeconomic markers.

Associations between disparities with selected macro development indicators were all weak, and statistically non-significant. Thus, unlike overall levels of child survival and other health indicators, higher income or socioeconomic development did not seem to reduce common disparities in child health.

This evidence calls into question the global strategies adopted so far to improve child health in the Arab region. Unlike in the 1970s and 1980s, there was currently no 'magic bullet' to improve child survival [63], and mother's education may no longer impact infant survival in urban areas [64]. Also, we know that economic growth or development may not be sufficient in generating health improvement of a population [65]. The evidence presented here pertaining to child health, rather than merely his/her survival, has important implications for social and health policy at the regional and national levels. Policies which aim to improve access to public and private health services and to reduce gender and economic disparities may help improve children's health status and wellbeing [56,66,67]. Given current levels of infant mortality, greater attention should be directed at broader issues of child wellbeing, and to the struggle for more equity in health care provision.

The analysis reported in this paper has some limitations. First, there were some gaps in the data available to us from these survey reports. Estimates for the child health indicators used were not available for all 18 countries. Furthermore, mortality rates by maternal education were available for only a small number of countries, preventing us from conducting meaningful analysis of socioeconomic differentials in mortality. Second, the reliability of the estimates reported varied, depending on the survey design and competence of the field organizations responsible for data collections. Third, although the rates provided by these surveys were more accurate than the estimates and projections used by UN agencies, the surveys' raw data files were not available to us for undertaking more in-depth analysis. Thus, we were not able to examine disparities at the household level, incorporating relevant risk factors into the analysis. It is of particular interest, for example, to examine gender disparities in child health within socioeconomic strata, but such detailed tabulations were not available to us. Also, disaggregated child health indicators by socioeconomic variables such as income or household wealth were lacking. Finally, the conclusions regarding the links between disparities and socioeconomic development across countries were based on bivariate analysis owing to the small sample used here. More analytic research using multi-level statistical techniques incorporating both household and country level effects using micro data is needed, but must wait 'data democratization' by countries in the region.

## Competing interests

The authors declare that they have no competing interests.

## Authors' contributions

MK conceived of the study, supervised preliminary analysis and data compilation, conducted statistical analysis, and participated in writing the final draft. JD prepared the data, conducted preliminary analysis, and drafted sections of the first draft. SMN and RY conducted literature review and participated in analysis and drafting sections of the final manuscript. All authors reviewed and approved the final manuscript.
